# Clinical Factors and Perinatal Outcomes Associated With Short Latency Period in Twin Pregnancies With Preterm Premature Rupture of Membranes Before 34 Weeks: A Retrospective Study

**DOI:** 10.3389/fmed.2022.839240

**Published:** 2022-03-04

**Authors:** Shuwei Zhou, Lingwei Mei, Wei Zhou, Yajun Yang, Xiaoyan Zhang, Xiaoling Mu, Quan Quan, Lan Wang

**Affiliations:** ^1^Department of Obstetrics, Chongqing Health Center for Women and Children, Chongqing, China; ^2^Department of Gynecology, The First Affiliated Hospital of Chongqing Medical University, Chongqing, China

**Keywords:** twin pregnancies, preterm premature rupture of membranes, latency period, clinical factors, perinatal outcomes

## Abstract

**Background:**

There is a lack of literature on short latency period (SLP) in twin pregnancies with preterm premature rupture of membranes (PPROM). Thus, the aim of this study was to identify the clinical factors and perinatal outcomes associated with SLP in twin pregnancies with PPROM and to establish a predictive model to identify SLP.

**Methods:**

Twin pregnancies with PPROM between 24 0/7 and 33 6/7 weeks were included and a retrospective analysis was performed. Patients were divided into two groups based on the latency period after PPROM: Group 1 ≤24 h (defined as SLP) and Group 2 >24 h (defined as long latency period, LLP), the clinical factors and perinatal outcomes were compared between the two groups. Binary logistic regression and receiver operating characteristic curve analyses were used to identify the independent clinical factors associated with latency period after PPROM and assess the predictive accuracy for SLP.

**Results:**

98 and 92 pregnant women had short and long latency period, respectively. Prolonged latency significantly increased the occurrence of chorioamnionitis. Neonatal outcomes were not affected by latency duration after PPROM. Binary regression analysis revealed that higher gestational age (GA) at PPROM (*P* = 0.038), presence of uterine contractions (*P* < 0.001), Bishop score > 4 (*P* = 0.030), serum procalcitonin levels ≥0.05 ng/mL upon admission, and absence of use of tocolytic agents (*P* < 0.001) were significant independent predictors of a SLP. A predictive model developed using these predictors had an area under the curve (AUC) of 0.838, and the presence of uterine contractions alone had an AUC of = 0.711.

**Conclusion:**

Uterine contraction was the most important prognosticator for a SLP. A latency period of >24 h was associated with chorioamnionitis, but adverse neonatal outcomes were not observed.

## Introduction

Preterm premature rupture of membranes (PPROM) occurs in 3% of all pregnancies, and approximately one-third of premature births are caused by or are related to PPROM, which is an important factor in neonatal morbidity and mortality (1–4). With the widespread use of assisted reproductive technology and ovulation induction, the rate of twin pregnancies has gradually increased. PPROM and preterm birth are more common in twin pregnancies; the incidence of PPROM in twin pregnancies is 7–10%, while it is only 2–4% for singleton pregnancies (3, 5, 6). The high incidence of PPROM in twins may mean that the mechanism is different from that in singletons, which may be related to uterine over-distension, increased intrauterine volume, cervical insufficiency, or elevated levels of sex hormones in twin pregnancies. Compared with singleton pregnancies, PPROM in twins tends to occur at an earlier gestational age (GA), and the time from membrane rupture to delivery is shorter (5, 7). However, there are no clinical practice guidelines specifically for PPROM in twin pregnancies, and management strategies only refer to singletons. The GA is the most important factor in determining neonatal outcomes after PPROM. In the absence of evidence of infection or other complications, expectant management is usually used to extend the GA as far as possible to reduce complications associated with preterm birth, and induced labor or cesarean section is performed at 34 weeks (3).

The latency period is defined as the time from rupture of membranes to delivery. There are many factors that affect the latency period after PPROM, but no risk factors have been identified, including the GA at PPROM, multiple pregnancies, oligohydramnios, cervical length, use of prophylactic antibiotics, and tocolysis (7–10). Multiple studies have shown that compared with singleton pregnancies, latency in twin pregnancies after PPROM was significantly shorter, with the median latency period being 3.6–4 days for twins and 6.2–7 days for singletons (11, 12). Twins were more likely to get delivered within 48 h (45.5% vs. 22.9%) or 72 h (11% vs. 4%) (6, 8). Twin pregnancies remained an independent risk factor for a short latency when controlling for other confounding factors (12, 13). In contrast, the results of a retrospective cohort study indicated that there was no statistical difference in the latency of twins and singletons in women presenting with PPROM (11). Additionally, a recent study has focused on identifying the clinical factors of latency period exceeding 48 h after PPROM (10), and a previous study modeled the latency period according to maternal and pregnancy characteristics, but these were mainly singleton pregnancies and only a small number of those with twins (14).

However, to date, there is a lack of information in the literature on the early recognition of short latency period (SLP) in twin pregnancies with PPROM. We have hardly found any research on infection indicators, such as serum inflammatory markers and vaginal microorganism culture that affected the latency period in twin pregnancies with PPROM.

In the present study, we aimed to identify clinical factors and perinatal outcomes of latency period within 24 h in twin pregnancies with PPROM before 34 weeks. Most previous studies were committed to exploring the clinical characteristics of singleton pregnancies, while ignoring the urgent healthcare needs of women with twin pregnancies. Identifying the significant predictors of a SLP in twin pregnancies with PPROM will provide a more appropriate clinical management and personalized treatment. Physicians could provide consultations, strengthen prenatal monitoring, and perform intrauterine transfers in time. The main purpose of this study was to identify the clinical factors associated with a SLP, to prolong the latency period, and to improve the perinatal outcomes in twin pregnancies with PPROM.

## Materials and Methods

### Participant Selection

This retrospective study reviewed the medical records of twin pregnant women with PPROM between 24 0/7 and 33 6/7 weeks who were admitted to the Obstetrics Department at Chongqing Health Center for Women and Children, a single tertiary referral center in Chongqing, China, from 1 January 2017 to 31 April 2021. We excluded women with any of the following conditions: at least one intrauterine death on admission, couples who gave up the pregnancy for induced labor, and with complications of monochorionic-diamniotic twins (for example, twin-to-twin transfusion syndrome and twin anemia polycythaemia sequence). The study was approved by the Ethics Committee of Chongqing Health Center for Women and children.

### Diagnostic Criteria and Definitions

#### PPROM Diagnostic Criteria

Rupture of membranes occurred at <37 weeks of gestation before labor. Diagnosis was based on pregnant women with a history of vaginal discharge, and on sterile speculum examination, leakage of amniotic fluid from the cervical os, or pooling of amniotic fluid in the posterior vaginal vault. If the characteristic findings were not significant, we performed an ultrasound examination to assess amniotic fluid volume, measured insulin-like growth factor binding protein-1, or conducted fern tests to assist in the diagnosis.

#### Determination of the GA

The GA was determined based on the first day of the woman's last menstruation and the crown-rump length measured by ultrasound in early pregnancy. GA was also estimated based on the date of embryo transfer when the pregnancy was conceived through assisted reproductive technology (ART).

#### Definition of Clinical Chorioamnionitis

Maternal body temperature ≥38°C accompanied by any of the following criteria can be diagnosed with clinical chorioamnionitis: malodorous vaginal discharge, fetal tachycardia (baseline ≥160 beats per minute), maternal tachycardia (≥110 beats per minute), maternal peripheral blood leukocyte count ≥ 15 × 10^9^/L, or the uterus is irritated with presence of uterine tenderness.

#### Management of PPROM

All patients of expectant management received dexamethasone (four intramuscular doses of 6 mg 12 h apart) for maturation of the fetal lungs and intravenous broad-spectrum prophylaxis (ampicillin, ceftezole, or clarithromycin) for 48 h, followed by oral amoxicillin and erythromycin for 5 days to prevent infection. Magnesium sulfate was routinely administered for fetal neuroprotective effects. Nifedipine, indomethacin, atosiban, and ritodrine were used as tocolytic agents depending on the status of the pregnant woman.

The status of the mother and fetus was closely monitored until delivery. Pregnant women underwent a vaginal examination on admission to obtain the Bishop score, and secretions were taken for microbial and group B streptococcus (GBS) cultures. The patients were checked daily by obstetricians. Maternal serum inflammatory markers, specifically blood routine examination, and C-reactive protein (CRP) and procalcitonin (PCT) levels were checked every 3 days. Ultrasound scans were performed every 3–4 days to evaluate the amniotic fluid volume and the status of the fetus. A single deepest pocket ≤ 2 cm was considered oligohydramnios. Non-stress test was initiated at 32 weeks daily to assess the status of the fetus and earlier GA for high risk patients.

When clinical chorioamnionitis was diagnosed, if premature birth was inevitable, if there were indications for delivery of the mother and/or fetus, or if GA reached 34 weeks, the pregnancy was terminated by induction of labor or cesarean section depending on the patient's desire, obstetric history and fetal status.

### Data Collection

We obtained maternal and fetal clinical information from patients' electronic medical records from the hospital information system. The database contains the following parameters: (1) Maternal parameters including the age, working condition, parity, history of preterm birth, and history of PPROM; (2) Information regarding current pregnancy including prepregnancy body mass index (BMI, kg/m^2^), mode of conception, chorionicity, GA at PPROM, maternal inflammatory markers (serum white blood cell count, neutrophil ratio and CRP, and PCT levels) before the administration of antibiotics or steroids, Bishop score [refer to the original text for (15)], uterine contractions, vaginal microbiological culture, GBS, use of tocolytic agents, pregnancy comorbidities (gestational diabetes mellitus, intrahepatic cholestasis of pregnancy, hypertensive disorder of pregnancy, cervical insufficiency); (3) Obstetric outcomes including clinical chorioamnionitis, placental abruption, cord prolapse, oligohydramnios, GA at delivery, mode of delivery, indication of termination of pregnancy, and latency period; (4) Fetal parameters including birth weight, neonatal death, admission to the neonatal intensive care unit (NICU), and major neonatal complications (respiratory distress syndrome, neonatal sepsis, intraventricular hemorrhage, necrotising enterocolitis, respiratory distress syndrome, bronchopulmonary dysplasia, and retinopathy of prematurity.

Pregnant women with PPROM were categorized into two groups based on the latency period. Patients in Group 1 had a latency period of ≤ 24 h, whereas those in Group 2 had a latency period of >24 h. Clinical factors and perinatal outcomes were compared between the two groups.

### Statistical Analyses

Data were analyzed using SPSS version 25.0 (IBM, Armonk, NY, USA). Descriptive statistics were used to analyse the patients' clinical factors and perinatal outcomes, and the chi-squared test or Fisher's exact test was used to analyse categorical variables. After assessing normality, the statistical significance of the continuous variables was assessed using the Student's *t*-test or the Mann-Whitney U test, and was also summarized as the mean ± standard deviation or the median with interquartile range. Following univariate analysis, binary logistic regression was performed to identify the independent factors of a SLP. Odds ratios (OR) and 95 % confidence intervals (CI) were calculated. Receiver operating characteristic (ROC) curves were used to assess the predictive accuracy of the SLP with twin pregnancies complicated by PPROM. *P* ≤ 0.050 was considered as statistically significant.

## Results

### Patient Characteristics

A total of 190 women with twin pregnancies (including 164 dichorionic diamniotic twins and 26 monochorionic diamniotic twins) with PPROM between 24 0/7 weeks and 33 6/7 weeks were included in the final analysis, of which 98 (51.6%) patients were in Group 1 and 92 patients (48.4 %) in Group 2. The mean maternal age of these patients was 30.3 ± 3.7 years. The median GA at PPROM was 32 1/7 weeks (Q1:30 0/7 weeks to Q3:33 1/7 weeks), and the median latency period was 22 hours (Q1:7 to Q3:71 h).

The clinical factors of short and long latency periods are described in Table 1. The maternal age, working condition, primipara, *in-vitro* fertilization conception, chorionicity, prepregnancy BMI, maternal inflammatory markers (except PCT), the positive rate of vaginal microbiological and GBS, and presence of pregnancy complications were similar between the two groups. Pregnant women with a SLP were more likely to have a high GA at PPROM, a Bishop score >4, serum PCT levels ≥0.05 ng/mL, uterine contractions upon admission, and not received tocolytic agents.

**Table 1:** Comparison of clinical factors associated with short latency period.

**Factors**	**Group 1** **(*n* = 98)**	**Group 2** **(*n* = 92)**	**Overall**	***P*-value**
Latency period (hours)	7 (5–12.5)	72.5 (46.5–147.5)	22 (7–70.5)	**<0.001**
Maternal age (year)	30.0 ± 3.2	30.5 ± 4.2	30.3 ± 3.7	0.362
At work	50 (51%)	45 (48.9%)	95 (50%)	0.772
Primipara	87 (88.8%)	80 (87%)	167 (87.9%)	0.701
History of PPROM or preterm birth	0 (0%)	1 (1.1%)	1 (0.5%)	0.484
Prepregnancy BMI (Kg/m^2^)	21.4 (19.0–23.6)	21.1 (19.4–23.1)	21.3 (19.2–23.6)	0.870
GA at PPROM (weeks)	32 3/7 (31 0/7–33 3/7)	31 4/7 (29 3 /7–32 6/7)	32 1 /7 (30 0/7–33 1/7)	**0.001**
White blood cell (*10^9^/L)	9 (7–11)	9 (8–11)	9 (7–11)	0.622
Neutrophil ratio (%)	77.5 ± 8.4	78.2 ± 6.8	77.8 ± 7.6	0.519
C-reactive protein (mg/L)	5 (2–13)	5 (1–10)	5 (2–11)	0.401
Procalcitonin ≥0.05 ng/mL	50 (57.5%)	37 (42%)	87 (49.7%)	**0.041**
Conception by IVF	72 (73.5%)	69 (75.0%)	141 (74.2%)	0.810
Dichorionic diamniotic	86 (87.8%)	78 (84.8%)	164 (86.3%)	0.551
Bishop score >4	64 (65.3%)	25 (27.2%)	89 (46.8%)	**<0.001**
Presence of contraction	69 (70.4%)	26 (28.3%)	95 (50.0%)	**<0.001**
Use tocolytic agent	14 (14.3%)	45 (48.9%)	59 (31.1%)	**<0.001**
Positive GBS	5 (10.2%)	1 (1.8%)	6 (5.7%)	0.064
Positive vaginal microbial	13 (21.7%)	21 (24.4%)	34 (23.3%)	0.699
Gestational diabetes mellitus	29 (29.6%)	22 (23.9%)	51 (26.8%)	0.377
ICP	13 (13.3%)	6 (6.5%)	(10.0%)	0.122
HDP	9 (9.2%)	5 (5.4%)	14 (7.4%)	0.323
Cervical insufficiency	5 (5.1%)	2 (2.2%)	7 (3.7%)	0.284

*Data are presented as n (%) or median (Interquartile, Q1–Q3) or mean ± standard deviation*.

*All unknown data were not included in the analysis*.

*PPROM, preterm premature rupture of membranes; BMI, body mass index; GA, gestational age; IVF, in vitro fertilization; GBS, group B streptococcus; ICP, intrahepatic cholestasis of pregnancy; HDCP, hypertensive disorder of pregnancy*.

### Binary Regression Analysis and Predictive Model of SLP

The final predictive model had five main factors, as listed in Table 2. In the binary logistic regression analysis to identify predictors of latency period, higher GA at PPROM (*P* = 0.020), and presence of uterine contraction (*p* < 0.001) demonstrated a significant independent association with a SLP. We also found that a Bishop score > 4 lowered the OR of the long latency period, and the use of tocolytic agents increased the OR of the SLP. Our model did not demonstrate an independent association of PCT ≥0.05 ng/mL with SLP.

**Table 2:** Binary logistic regression models of short latency period.

**Factors**	**OR (95% CI)**	**Comparison**	***P*-value**
GA at PPROM (days)	1.03 (1.00–1.05)	continuous	**0.038**
Uterine contraction	5.57 (2.26–13.75)	Present ref absent	**<0.001**
Bishop score	2.67 (1.10–6.48)	>4 ref ≤ 4	**0.030**
Use of tocolytic agent	6.50 (2.68–15.79)	Present ref absent	**<0.001**
Serum PCT (ng/mL)	2.03 (0.96–4.30)	≥0.05 ref <0.05	0.066

*Ref, reference; OR, odds ratio; CI, confidence interval; GA, gestational age; PPROM, preterm premature rupture of membranes; PCT, procalcitonin*.

*OR > 1 indicates improved odds of short latency period relative to the comparison group*.

**Figure 1:**
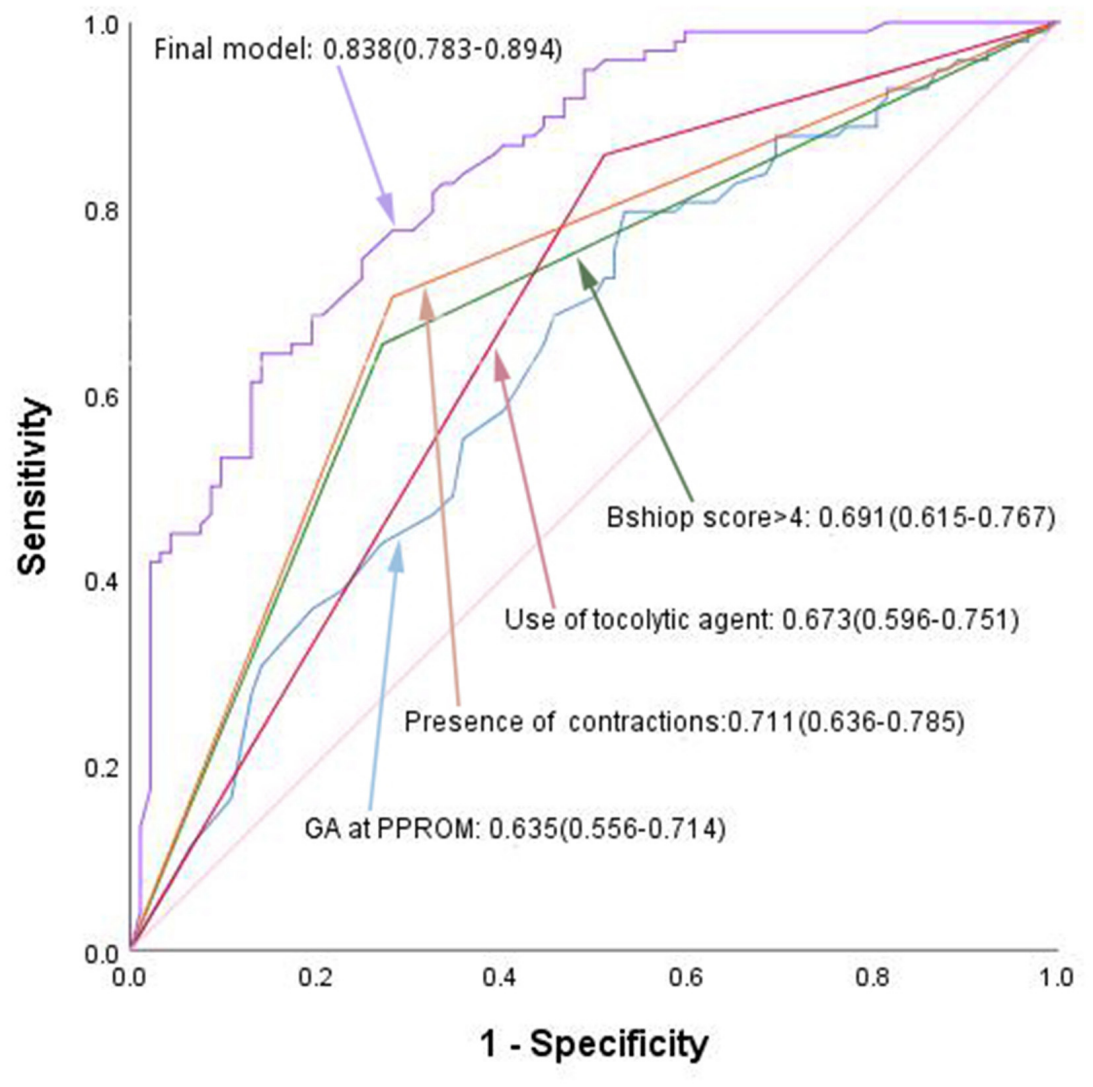
ROC curves of the final predictive model, compared to separate independent predictor. GA, gestational age; PPROM, preterm premature rupture of membranes.

The predictive accuracy of the final model was assessed using the area under the AUC curve. The final model using these independent variables had an AUC of 0.838, as shown in Figure 1. Among the four clinical features of independent prediction, the presence of uterine contraction predicted a short latency period with the highest accuracy (AUC = 0.711), and the other three factors alone were not very strong in predicting SLP (AUC = 0.635–0.691).

### Perinatal Outcomes

We examined differences in perinatal outcomes according to latency period (Table 3). A latency period of ≥24 h was associated with an increased risk of chorioamnionitis, but there was no significant difference in placental abruption, cord prolapse, oligohydramnios, the GA at delivery, and the mode of delivery between the two groups. The indications for termination of pregnancy in Group 1 were mostly preterm labor (70.4%), followed by maternal or fetal complications, and none of the pregnancies lasted until 34 weeks. In addition, the median birth weight was high in patients with a SLP (*p* = 0.02). Twenty-three fetuses (6.1%) experienced intrauterine demise or neonatal death in total, independent of the latency period. All statistical neonatal outcomes (except birth weight) between the two groups were not statistically significant.

**Table 3:** Perinatal outcomes associated with PPROM according to latency period.

**Factors**	**Group 1** (***n =* 98/196)**	**Group 2** **(*n =* 92/184)**	**Overall**	***P*-value**
**Obstetric outcomes**				
GA at delivery (weeks)	32 4/7 (31 1/7–33 3/7)	32 3/7 (29 6/7–33 3/7)	32 3/7 (30 3/7–33 /7)	0.566
Placental abruption	6 (6.1%)	4 (4.3%)	10 (5.3%)	0.748
Cord prolapse	6 (6.1%)	2 (2.2%)	8 (4.2%)	0.176
Oligohydramnios	19 (19.4%)	23 (25.0%)	42 (22.1%)	0.352
Clinical chorioamnionitis	6 (6.1%)	15 (16.3%)	21 (11.1%)	**0.025**
Cesarean section	86 (87.8%)	80 (87.0%)	166 (87.4%)	0.868
Indications for termination				**<0.001**
Preterm labor	69 (70.4%)	45 (48.9%)	114 (60.0%)	
Complications	29 (29.6%)	38 (41.3%)	67 (35.3%)	
Pregnancy lasted 34 weeks	0 (0%)	9 (9.8%)	9 (4.7%)	
**Neonatal outcomes**				
Birth weight (Kg)	1.80 (1.55–2.00)	1.65 (1.34–1.93)	1.74 (1.43–1.95)	**0.020**
Foetal or neonatal death	8 (4.1%)	15 (8.2%)	23 (6.1%)	0.096
Admission to NICU	54 (28.7%)	54 (32.0%)	108 (30.3%)	0.507
Neonatal sepsis	36 (19.1%)	42 (24.9%)	78 (21.8%)	0.193
Respiratory distress syndrome	64 (34.0%)	53 (31.4%)	117 (32.8%)	0.590
Necrotizing enterocolitis	14 (7.4%)	22 (13.0%)	36 (10.1%)	0.081
Intraventricular hemorrhage	15 (8.0%)	12 (7.1%)	27 (7.6%)	0.754
Retinopathy of prematurity	22 (11.7%)	20 (11.8%)	42 (11.7%)	0.969
Bronchopulmonary dysplasia	12 (6.4%)	15 (8.9%)	27 (7.6%)	0.374

*The result of neonatal complications excluded dead fetus and newborns*.

*GA, gestational age; NICU, neonatal intensive care unit*.

## Discussion

Recently, substantial studies have highlighted the clinical factors associated with a LLP (defined latency period ≥48, 72 h, 7 days, or even 14 days) among singleton pregnancies with PPROM (7, 9, 10). There are also studies that focus on comparing the latency period of PPROM between twin and singleton pregnancies (5, 12), but few have individually identified these characteristics of SLP in twin pregnancies with PPROM. In this study, we identified clinical factors associated with a latency period of ≤ 24 h in twin pregnancies with PPROM before 34 weeks of gestation. Our results revealed that patients who were considered to have a SLP were prone to have the following characteristics: a high GA at PPROM, presence of uterine contractions, Bishop score >4, serum PCT levels ≥0.05 ng/mL, and absence of use of tocolytic agents in univariate analyses. This finding is significant because it might identify a SLP upon admission and transfer pregnant women to appropriate hospitals in time to attempt to receive novel therapeutic regimens and improve perinatal outcomes.

The standpoint that PPROM in twin pregnancies tends to have a SLP than singleton pregnancies is widely recognized (5, 12–14). A recent study has shown that the median latency period was 4 days in twins after PPROM (12), and a Chinese study indicated that half of pregnant women deliver 2 days after PPROM (13). In this study, the median latency period of twin pregnancies with PPROM was 22 h, which was shorter than that reported in other studies (5, 12, 13). The main reason is that the median GA at PPROM was 32 1/7 weeks in this study, which is negatively correlated with the latency period; however, the mean GA at PPROM was 29.1 weeks and at delivery was 29.3 weeks in other studies (5, 12). Furthermore, in a study of 49 twin pregnancies, the median GA at PROM was 31 weeks, and the median latency period was 0 days, which is similar to our observations (16). Our results are consistent with majority of studies, which have shown an inverse correlation between the latency period and GA at PPROM (9, 13, 17). However, other studies have failed to observe any correlation between the latency period and GA (10, 18).

When controlling for other confounding factors, we identified four significant predictors of a SLP in a multivariate analysis. The predictive model (AUC, 0.838) may adequately evaluate SLP at admission to change traditional treatments that might eventually improve perinatal outcomes. In summary, there are multiple avenues to experience a SLP, but the strongest factor may be the presence of uterine contractions (alone AUC, 0.711), while early and high doses of tocolytic agents may prolong the latency period. A similar correlation was found in the study by Phupong et al., who found that prophylactic tocolysis was a major factor associated with a latency period ≥2 days after PPROM (9). Some scholars have reported that the need for tocolysis can predict a SLP (10). The reason for this difference is that the time of tocolysis was different among the studies. In the former study, it was administered before uterine contractions, and in the latter, after contractions. In our institution, tocolytic agents are used to suppress uterine contractions instead of being routinely administrated. The indication of termination of pregnancy in most patients who delivered within 24 h after PPROM was premature labor. Therefore, based on the above research results, prophylactic tocolysis may prolong the latency period.

Recent data indicated that cervical length and cervical dilation at admission were inversely associated with the latency period in twin preganncies complicated by PPROM (17, 19). However, to date, there has been almost no report on the relationship between the Bishop score and latency period. The Bishop score may currently be the best tool for assessing cervical status, and it is a good predictor of vaginal delivery (15, 20). Our data suggest that a SLP was more likely to have a Bishop score >4, and more research is needed to explore the relationship between the Bishop score and latency period in PPROM.

Intraamniotic infection has been associated with the initiation and progression of PPROM (21). High maternal serum inflammatory markers, such as leukocytes, CRP, and PCT, may be risk factors for intrauterine infection. Asadi et al. suggested that CRP had high accuracy in predicting chorioamnionitis in PPROM and found that patients diagnosed with chorioamnionitis had significantly high serum levels of CRP both on admission and before termination of pregnancy (22). A prospective study indicated that maternal serum PCT level used 0.054 ng/mL as the cut-off value for the prediction of histological chorioamnionitis had high specificity and sensitivity in PPROM (23). Few previous studies have investigated the effect of inflammatory markers on the latency period after PPROM in twin pregnancies.…. In this study, we discovered that the latency period within 24 h after PPROM was more be inclined to have a high maternal serum PCT level (>0.05 g/mL) in univariate analysis; however, the correlation disappeared after controlling for other confounding factors and that no correlation was observed between other inflammatory markers and the latency period. In general, the value of maternal PCT determination in the diagnosis of latency period is unsatisfactory in twin pregnancies with PPROM (16). Further prospective and large sample research is needed to investigate the role of inflammatory markers in the latency period after PPROM.

Our study demonstrates that the occurrence of chorioamnionitis is related to prolonged latency. An inseparable relationship between PPROM and chorioamnionitis has been confirmed in numerous studies (7, 8, 24, 25). Obstetricians terminate the pregnancy when combined with clinical chorioamnionitis, thus shortening the time from rupture of membranes to delivery (24). On the contrary, pregnant women showing prolonged latency periods are at an increased risk of chorioamnionitis, as we observed (7, 8). This is due to a prolonged latency allowing microorganisms more time to enter the uterine cavity after PPROM, leading to an increase in the chance of infection (26). Therefore, it is necessary to closely monitor clinical signs and inflammatory markers and administer antibiotics appropriately after PPROM.

We found that prolonged latency was unrelated to perinatal outcomes, Unlike chorioamnionitis, neonatal sepsis was not related to the duration of latency; this finding has also been confirmed by several other studies (27–29). Two studies revealed that a prolonged latency period was associated with a decreased incidence of neonatal mortality or morbidity (13, 29). There are also research reported neonatal outcomes were not affected by latency duration after PPROM (7, 10, 28). Compared with singleton pregnancies with PPROM, twins were less likely to be complicated by chorioamnionitis or placental abruption (5, 11), but tend to have adverse neonatal outcomes (12). In addition, we observed that newborns who were delivered within 24 h after PPROM had an increased birth weight, which is different from the findings of other studies (7, 12), which may be because the GA at PPROM was high.

Indeed, some study population have been reported in our previous article (30), and the main purpose of which was to describe perinatal outcomes of twin pregnancies with PPROM. However, there is no overlap in reported data since the parameters related to latency period was not analyzed in previous paper. This study is the first to explore the clinical factors and perinatal outcomes of SLP in twin pregnancies with PPROM and has a large sample size. This is also the first study to investigate the relationship between GBS, vaginal microbiological culture, and latency period in twins, although the results were not statistically different. Our study had two limitations. First, this was a retrospective study, and potential confounding variables could not be ruled out; prospective randomized studies with adequate cases should be performed to obtain more reliable results. Second, only a small number of placentas underwent histopathological examination, and the association between the latency period and histological chorioamnionitis could not be investigated. Further prospective studies should focus on the associations revealed by this analysis.

## Conclusion

The present study provides evidence to identify a SLP according to clinical factors. Our study showed that a high GA at PPROM, presence of uterine contractions, Bishop score>4, and absence of use of tocolytic agents were associated with a shortened latency period in twin pregnancies with PPROM. The most important prognosticator was the presence of uterine contraction. A latency period >24 h was associated with chorioamnionitis, but adverse neonatal outcomes were not observed. Accordingly, the data presented in this study can be used by obstetricians to provide consultation and provide predict the latency period for the ease of intrauterine transfer and personalized treatment. Additionally, more studies are needed to achieve a reliable and feasible model for clinically useful calculations.

## Data Availability Statement

The original contributions presented in the study are included in the article/supplementary material, further inquiries can be directed to the corresponding author/s.

## Ethics Statement

The studies involving human participants were reviewed and approved by The Ethics Committee of Chongqing Health Center for Women and Children. Written informed consent for participation was not required for this study in accordance with the national legislation and the institutional requirements.

## Author Contributions

SZ, LM, and LW designed the study. SZ and LM drafted the manuscript. XM and QQ conducted the statistical analysis. YY and SZ collected the data. LW and WZ revised the manuscript. All authors have read and approved the final study.

## Funding

This study was founded by Chongqing Health Commission and Science and Technology Bureau (2019GDRC013). The role of this funding is in editing the manuscript and article-processing charge.

## Conflict of Interest

The authors declare that the research was conducted in the absence of any commercial or financial relationships that could be construed as a potential conflict of interest.

## Publisher's Note

All claims expressed in this article are solely those of the authors and do not necessarily represent those of their affiliated organizations, or those of the publisher, the editors and the reviewers. Any product that may be evaluated in this article, or claim that may be made by its manufacturer, is not guaranteed or endorsed by the publisher.
